# Infection is an Independent Predictor of Death in Diffuse Large B Cell Lymphoma

**DOI:** 10.1038/s41598-017-04495-x

**Published:** 2017-06-30

**Authors:** Claire Dendle, Michael Gilbertson, Tim Spelman, Rhonda L. Stuart, Tony M. Korman, Karin Thursky, Stephen Opat, Zoe McQuilten

**Affiliations:** 1 School of Clinical Sciences, Monash University, Wellington Road, Clayton, VIC 3800 Australia; 2 Monash Infectious Diseases, Level 3, Monash Medical Centre, 246 Clayton Road, Clayton, VIC 3168 Australia; 3 Monash Haematology, Monash Health, Level 4, Monash Medical Centre, 246 Clayton Road, Clayton, VIC 3168 Australia; 4 Centre for Population Health, Burnet Institute, Melbourne, Australia; 5 Department of Infectious Diseases, Peter MacCallum Cancer Centre, 305 Grattan Street, Melbourne, VIC 3000 Australia; 6 Department of Medicine, University of Melbourne, Parkville, VIC 3010 Australia; 7 Department of Epidemiology and Preventive Medicine, Monash University, Commercial Road, Melbourne, VIC 3004 Australia

## Abstract

To identify risk factors for infection in patients with diffuse large B cell lymphoma (DLBCL) undergoing rituximab, cyclophosphamide, vincristine, adriamycin and prednisolone (R-CHOP) treatment. All patients with DLBCL who received R-CHOP from 2004–2014 in a tertiary Australian hospital were identified and information collected from hospital admission data, laboratory results and medical record review. Infection was defined as hospitalisation with an ICD-10-AM diagnostic code for infection. Risk factors for infection and association between infection and survival were modelled using Cox proportional hazards regression. Over the 10-year period there were 325 patients; 191 (58.8%) males, median age 66 years. 206 (63.4%) patients experienced ≥1 infection. Independent predictors of infection were Charlson comorbidity index score (hazard ratio [HR] 3.60, p = 0.002), Eastern Cooperative Oncology Group (ECOG) performance status (HR 2.09 p = <0.001) and neutropenia (HR 2.46, p = <0.001). 99 (31%) patients died. Infection was an independent predictor of survival (HR 3.27, p = <0.001, as were age (HR 2.49, p = 0.001), Charlson comorbidity index (HR 4.34, p = <0.001), ECOG performance status (HR 4.33, p = 0.045) and neutropenia (HR 1.95, p = 0.047). Infections are common and infection itself is an independent predictor of survival. Patients at highest risk of infection and death are those with multiple comorbidities, poor performance status and neutropenia.

## Introduction

Non-Hodgkin Lymphoma is one of the most common adult malignancies^1^ and diffuse large B cell lymphoma (DLBCL) the most frequent histological subtype^2^. Treatment with rituximab, cyclophosphamide, doxorubicin, vincristine and prednisolone (R-CHOP) is currently standard of care for DLBCL, with three year overall survival ranging from 50 to >95% depending upon prognostic variables^3^. Infection is a common cause of morbidity and mortality with neutropenic fever occurring in 10–20% of patients treated for lymphoma^4–13^. However there is limited information on the risk factors and impact of infection among patients treated for DLBCL. The ability to define a high-risk subset of patients may be useful for targeted application of preventative therapies.

The aim of this study was to determine the incidence, risk factors and timing of infections in patients with DLBCL treated with R-CHOP and R-CHOP-like chemotherapy, and to explore the association between infection and overall survival.

## Results

### Description of patient cohort

Over the 10-year period there were 325 patients with DLBCL who received R-CHOP or R-CHOP-like chemotherapy with curative intent. Median follow up of surviving patients was 2.54 years (IQR 1.11, 4.93).

Demographic details are outlined in Table 1. There were 191 (58.8%) males and the median age at diagnosis was 67.0 years. The most common Charlson comorbidity score was 0–2 in 270 (83.1%) and the most common ECOG status at diagnosis was 1 in 111 (38.5%). The most common stage at diagnosis was stage IV in 150 (48.4%). The median number of R-CHOP chemotherapy cycles was six. 112 (34.5%) had a raised creatinine at baseline.

**Table 1:** DEMOGRAPHICS.

Characteristic		Number	Percent
Age in years (n = 325)		66	IQR −55.8–77.1
Sex (n = 325)	Female	134	41.3
	Male	191	58.8
Charleson comorbidity score (n = 325)	0–2	270	83.1
	3–5	48	14.8
	6+	7	2.2
ECOG status (n = 288)	0	73	25.4
	1	111	38.5
	2	81	28.1
	3	22	7.6
	4	1	0.4
Stage (n = 310)	1	32	10.3
	2	86	27.7
	3	42	13.6
	4	150	48.4
NCCN IPI (n = 315)	Low risk	26	8.0
	Low intermediate	97	29.9
	High Intermediate	113	34.8
	High	79	24.3
Chemotherapy type (n = 325)	R-CHOP 21	286	90.8
	R-CHOP 14	20	6.2
	R-CEOP	4	1.2
	R-CVP	3	0.9
	R-CODOXM/IVAC	3	0.9
	Other combinations of rituximab, cyclophosphamide, vincristine or prednisolone	9	2.7
No. of chemotherapy cycles (n = 316)	1	6	1.9
	2	14	4.3
	3	16	4.9
	4	37	11.4
	5	9	2.8
	6	219	67.4
	>6	15	4.7
Creatinine (n = 320)	Normal	208	64.0
	Raised	112	34.5

ECOG = Eastern Cooperative Oncology Group performance status point scale.

NCCN-IPI = International Prognostic Index.

R-CHOP-21: rituximab, cyclophosphamide, doxorubicin, vincristine and prednisolone administered every 21 days.

R-CHOP-14: rituximab, cyclophosphamide, doxorubicin, vincristine and prednisolone administered every 14 days.

R-CEOP: rituximab, cyclophosphamide, etoposide, vincristine and prednisolone.

R-CVP: rituximab, cyclophosphamide, vincristine and prednisolone.

R-CODOXM/IVAC: rituximab, cyclophosphamide, doxorubicin, vincristine and prednisolone, cytarabine, methotrexate.

### Description of infections

206 patients (63.4%) patients experienced at least one infection with a single admission in 82 patients (25.2%), two in 50 (15.4%), three in 32 (9.9%) and four or more presentations in 42 (12.9%). Of the 206 patients with an infection, 25 (3.7%) required ICU admission and 19 (2.8%) required mechanical ventilation. The median time from the first day of chemotherapy to first infection was 85 days (IQR 52–134).

Overall, there were 3732 admissions recorded with 517 (13.9%) infections. The site of infection was recorded in 322 (62.3%) (Table 2). The most common sites of infection were lower respiratory tract (40.7%), skin and soft tissue (18.7%) and blood stream infection (15.1%).

**Table 2:** SITES OF INFECTION – All infectious episodes (n = 332).

Blood stream infection	50 (15.1%)
Lower respiratory tract	135 (40.7%)
Upper respiratory tract	30 (9.0%)
Cardiovascular	8 (2.4%)
Gastrointestinal	6 (1.8%)
Urogenital	6 (1.8%)
Neurological	3 (0.9%)
Skin and Soft tissue	62 (18.7%)
Bone and Joint	2 (0.6%)
Other	1 (0.3%)
Device or line related	24 (7.2%)

A diagnostic code specifying a microbiological organism was reported in 375 (72.5%) of the 517 infection episodes. Bacteria accounted for 186 (49.6%), viruses for 117 (31.2%) and fungi for 72 (19.2%). Within the bacterial category, there were 50 (13.6%) blood stream isolates. Of the blood stream isolates, gram-negative bacteria were the most common isolates, accounting for 39 (10.6%) and *E. coli* was the most frequently isolated gram-negative blood stream isolate. Of the blood stream isolates, *Staphylococcus* species and *Streptococcus* species accounted for 10 (2.7%) and 1 (0.3%), respectively. Of all bacterial isolates, gram-negative bacteria accounted for 99 (27.0%), gram-postive for 47 (12.8%) and other bacteria for 33 (9%). There were seven patients with* Mycobacterium* species, five (1.4%) with tuberculosis and two (0.5%) with other mycobacteria. Of the viral isolates, herpes viruses accounted for 20 (17.1%), hepatitis B for 49 (41.9%), hepatitis C for 38 (32.5%) and HIV for 2 (1.7%). Within the fungal category, *Candida* species accounted for 59 (81.9%) however 39 (54.1%) were oral candidiasis. *Aspergillus* species for 2 (2.7%), other fungi for 2 (2.7%) and *Pneumocystis jirovecci* for 9 (12.5%) infections (Table 3).

**Table 3:** MICROBIOLOGICALLY CONFIRMED INFECTIONS n = 375.

Class of organism	Organism	Number of isolates n (%)
Bacterial (Gram-positive organisms) 47	*Staphylococcus aureus*	11 (2.9)
	*Coagulase negative Staphylococcal spp*.	25 (6.7)
	*Streptococcus pneumoniae*	1 (0.3)
	*Other Streptococcal spp*.	10 (2.7)
Bacterial (Gram-negative organisms)	*Escherichia coli*	15 (4.0)
	*Klebsiella spp*.	12 (3.2)
	*Pseudomonas spp*.	12 (3.2)
	*Campylobacter spp*.	3 (0.8)
	Other gram negative bacteria	57 (15.2)
Bacterial other	Bacteria other	19 (5.1)
	*Clostridium difficile*	14 (3.7)
Mycobacterial	*Mycobacterium tuberculosis*	5 (1.3)
	*Mycobacterium spp*. other	2 (0.5)
Viral	*Herpes spp*.	10 (2.7)
	*Varicella zoster virus*	10 (2.7)
	Hepatitis B	49 (13.1)
	Hepatitis C	38 (10.1)
	HIV	2 (0.5)
	Influenza	3 (0.8)
	Viral infection other	5 (1.3)
Fungal	*Candida spp*.	59 (15.7)
	*Aspergillus spp*.	2 (0.5)
	Other fungal spp.	2 (0.5)
	*Pneumocystitis jirovecci*	9 (2.4)

Neutropenia was identified in 218 (5.8%) of 3732 admissions. Of admissions in which the patient was neutropenic, 59 (27.1%) had an associated infection code compared with 253 (11.3%) of 2227 (59.8%) admissions where the patient was not neutropenic.

Prior administration of pegfilgrastim was identified in 1439 (38.5%) admissions. Of those patients who received pegfilgrastim, 141 (9.7%) had an associated infection code compared to 376 (16.4%) of 2293 (62.5%) patients who did not receive pegfilgrastim.

### Predictors of an infectious episode

The results of the regression analysis of factors associated with an infectious episode are shown in Table 4.

**Table 4:** Regression analysis of the factors associated with infection in all study patients (n = 325).

		Univariate analysis		Multivariable analysis	
		Hazard Ratio	95% CI p value	Hazard Ratio	95% CI p value
Age	<65 years	1.00		1.00	
	>65 years	1.23	1.02 to 1.47 p = 0.025	0.96	0.77 to 1.18 p = 0.69
Sex	Female	1.00		1.00	
	Male	0.97	0.81 to 1.17 p = 0.78	0.95	0.77 to 1.19 p = 0.69
Charlson Comorbidity Score	1–2	1.00		1.00	
	3–5	3.60	2.88 to 4.51 p = <0.001	2.16	1.71 to 2.74 p = <0.001
	6+	5.35	3.47 to 8.26 p = <0.001	3.91	2.43 to 6.28 p = <0.001
ECOG	0	1.00		1.00	
	1	2.44	1.77 to 3.37 p = <0.001	2.09	1.46 to 3.01 p = <0.001
	2	4.58	3.33 to 6.30 p = <0.001	3.33	2.22 to 5.04 p = <0.001
	3 and 4	5.95	3.89 to 9.10 p = <0.001	3.36	1.99 to 5.66 p = <0.001
Stage	1	1.00		1.00	
	2	1.69	1.11 to 2.58 p = 0.013	1.78	1.11 to 2.84 p = 0.017
	3	2.18	1.41 to 3.40 p = 0.001	1.88	1.12 to 3.17 p = 0.017
	4	2.22	1.49 to 3.30 p = <0.001	1.71	1.04 to 2.82 p = 0.36
NCCN IPI	Low risk	1.00		1.00	
	Low intermediate	2.87	1.51 to 5.47 p = 0.001	4.19	1.45 to 12.07 p = 0.008
	High intermediate	4.87	2.58 to 9.18 p = <0.001	3.99	1.29 to 12.34 p = 0.016
	High	5.47	2.88 to 10.41 p = <0.001	3.69	1.12 to 12.14 p = 0.032
Number of chemotherapy cycles	1–2	1.00			
	3–4	0.65	0.40 to 1.05 p = 0.081	1.21	0.71 to 2.04 p = 0.48
	5–6	0.62	0.40 to 0.96 p = 0.035	0.91	0.57 to 1.45 p = 0.69
	>6	1.22	0.73 to 2.05 p = 0.444	1.41	0.81 to 2.44 p = 0.22
Creatinine	Normal	1.00		1.00	
	Raised	1.60	1.31 to 1.90 p = <0.001	1.06	0.84 to 1.33 p = 0.64
Neutropenia within 48 hours of admission with infection	No	1.00		1.00	
	Yes	2.68	2.10 to 3.41 p = <0.001	2.46	1.91 to 3.17 p = <0.001
Pegfilgrastim w/I 21 days of admission with infection	No	1.00		1.00	
	Yes	0.60	0.40 to 0.74 p = <0.001	0.71	0.57 to 0.88 p = 0.002

ECOG = Eastern Cooperative Oncology Group performance status point scale.

NCCN-IPI = International Prognostic Index.

After adjustment for all other model covariates, factors which remained significant predictors of infection in the multivariable analysis included Charlson comorbidity score three or greater (reference category score of 2 or less), ECOG status of one, two, three or four (with zero the reference category), and NCCN-IPI low/intermediate or greater (reference category low). Neutropenia within 48 hours of admission was also associated with an increased risk of infection (compared with neutrophil count >1 × 10^9^/L within the 48 hours prior). The use of pegfilgrastim in the preceding 21 days was associated with a reduced risk of infection (compared with no use of pegfilgrastim in the preceding 21 days).

The regression analysis for predictors of infection was also performed including only patients who received R-CHOP on a 21 day cycle and excluding patients who received R-CHOP like therapy or R-CHOP on a 14 day cycle. See Table 5.

**Table 5:** Regression analysis of the factors associated with infection in patients who received R-CHOP 21 (n = 286).

		Univariate analysis		Multivariable analysis	
		Hazard Ratio	95% CI p value	Hazard Ratio	95% CI p value
Age	<65 years	1.00		1.00	
	>65 years	1.25	1.03 to 1.51 p = 0.023	0.52	0.74 to 1.16 p = 0.69
Sex	Female	1.00		1.00	
	Male	0.97	0.80 to 1.18 p = 0.81	1.00	0.81 to 1.24 p = 0.93
Charlson Comorbidity Score	1–2	1.00		1.00	
	3–5	3.55	2.79 to 4.52 p = <0.001	1.93	1.49 to 2.50 p = <0.001
	6+	5.65	3.49 to 9.12 p = <0.001	4.26	2.55 to 7.11 p = <0.001
ECOG	0	1.00		1.00	
	1	2.28	1.64 to 3.18 p = <0.001	1.95	1.33 to 2.86 p = 0.001
	2	4.30	3.09 to 5.99 p = <0.001	3.36	2.16 to 5.21 p = <0.001
	3 and 4	5.95	3.89 to 9.10 p = <0.001	3.36	1.99 to 5.66 p = <0.001
Stage	1	1.00		1.00	
	2	1.8	1.16 to 2.93 p = <0.001	1.37	0.82 to 2.29 p = 0.228
	3	2.49	1.54 to 4.02 p = <0.001	1.57	0.90 to 2.71 p = 0.110
	4	2.51	1.61 to 3.89 p = 0.001	1.36	0.79 to 3.17 p = 0.261
NCCN IPI	Low risk	1.00		1.00	
	Low intermediate	4.55	1.85 to 11.17 p = 0.001	3.12	1.24 to 7.85 p = 0.015
	High intermediate	7.88	3.24 to 19.16 p = <0.0001	3.06	1.14 to 8.20 p = 0.026
	High	8.95	3.65 to 21.94 p = <0.0001	3.83	0.99 to 8.09 p = 0.052
Number of chemotherapy cycles	1–2	1.00		1.00	
	3–4	0.60	0.32 to 1.11 0.106	1.02	0.53 to 1.93 p = 0.94
	5–6	0.67	.38 to 1.17 p = 0.163	0.77	0.43 to 1.37 p = 0.37
	>6	1.41	0.74 to 2.67 p = 0.284	1.23	0.64 to 2.44 p = 0.51
Creatinine	Normal	1.00		1.00	
	Raised	1.55	1.28 to 1.89 p = <0.0001	1.10	0.85 to 1.42 p = 0.48
Neutropenia within 48 hours of admission with infection	No	1.00		1.00	
	Yes	4.92	2.82 to 8.59 p = <0.0001	2.68	2.05 to 3.51 p = <0.0001
Pegfilgrastim w/I 21 days of admission with infection	No	1.00		1.00	
	Yes	0.67	0.44 to 0.69 p = <0.001	0.71	0.53 to 0.85 p = 0.001

ECOG = Eastern Cooperative Oncology Group performance status point scale.

NCCN-IPI = International Prognostic Index.

### Overall Survival

Over the 10 year study period, 99 (30.5%) of the 325 patients died. For those who died, the median time from diagnosis to death was 273 days (129–636 days). The cause of death was progressive lymphoma in 58 (58.6%), infection in 12 (12.1%), another cancer in five (5.5%), liver failure in four (4.4%), other in seven (7.1%) and unknown in 11 (11.1%).

The results of regression analysis of the factors associated with overall survival are shown in Table 6.

**Table 6:** Regression analysis of the factors associated with survival in all study patients (n = 325).

		Univariate analysis		Multivariable analysis	
		Hazard Ratio	95% CI p value	Hazard Ratio	95% CI p value
Age	<65 years	1.00		1.00	
	>65 years	2.50	1.64 to 3.82 p = <0.001	2.49	1.42 to 4.35 p = 0.001
Sex	Female	1.00		1.00	
	1.03	0.70 to 1.52 p = 0.887	1.01	0.66 to 1.56 p = 0.85	1.03
Charlson Comorbidity Score	1–2	1.00		1.00	
	3–5	4.54	2.70 to 7.63 p = <0.001	4.34	2.00 to 6.33 p = <0.001
	6+	11.26	5.77 to 21.97 p = <0.001	7.36	3.38 to 16.00 p = <0.001
ECOG	0	1.00		1.00	
	1	4.33	1.81 to 10.34 p = <0.001	2.61	1.02 to 6.66 p = 0.045
	2	8.47	3.56 to 20.13 p = <0.001	2.41	0.84 to 6.95 p = 0.10
	3 and 4	19.83	7.49 to 52.64 p = <0.001	7.16	2.04 to 25.06 p = 0.002
Stage	1	1.00		1.00	
	2	1.27	0.54 to 2.95 p = 0.583	1.70	0.68 to 4.30 p = 0.25
	3	1.59	0.64 to 3.94 p = 0.318	1.50	0.51 to 4.41 p = 0.45
	4	2.23	1.02 to 4.87 p = 0.046	1.90	0.71 to 5.10 p = 0.19
NCCN IPI	Low risk	1.00		1.00	
	Low intermediate	7.16	0.97 to 52.72 p = 0.053	2.56	0.31 to 20.76 p = 0.280
	High intermediate	9.66	1.32 to 70.44 p = 0.025	2.87	0.32 to 25.90 p = 0.34
	High	18.18	2.49 to 132.54 p = 0.004	4.27	0.43 to 42.38 p = 0.21
Number of chemotherapy cycles	1–2	1.00		1.00	
	3–4	0.18	0.10 to 0.34 p = <0.001	0.39	0.18 to 0.84 p = 0.016
	5–6	0.09	0.05 to 0.15 p = <0.001	0.13	0.06 to 0.25 p = <0.001
	>6	0.09	0.03 to 0.27 p = <0.001	0.10	0.03 to 0.36 p = <0.001
Creatinine	Normal	1.00		1.00	
	Raised	1.23	0.82 to 1.84 p = 0.318	0.31	0.43 to 1.30 p = 0.31
Neutropenia within 48 hours of admission with infection	No	1.00		1.00	
	Yes	4.45	2.61 to 7.60 p = <0.001	1.95	1.01 to 3.78 p = 0.047
Pegfilgrastim w/I 21 days of admission with infection	No	1.00		1.00	
	Yes	0.69	0.43 to 1.10 p = 0.124	0.8	0.58 to 1.65 p = 0.58
Admission with infection	No	1.00		No	1.00
	Yes	5.08	3.46 to 7.49 p = <0.001	3.27	2.03 to 5.27 p = <0.001

ECOG = Eastern Cooperative Oncology Group performance status point scale.

NCCN-IPI = International Prognostic Index.

**Figure 1:**
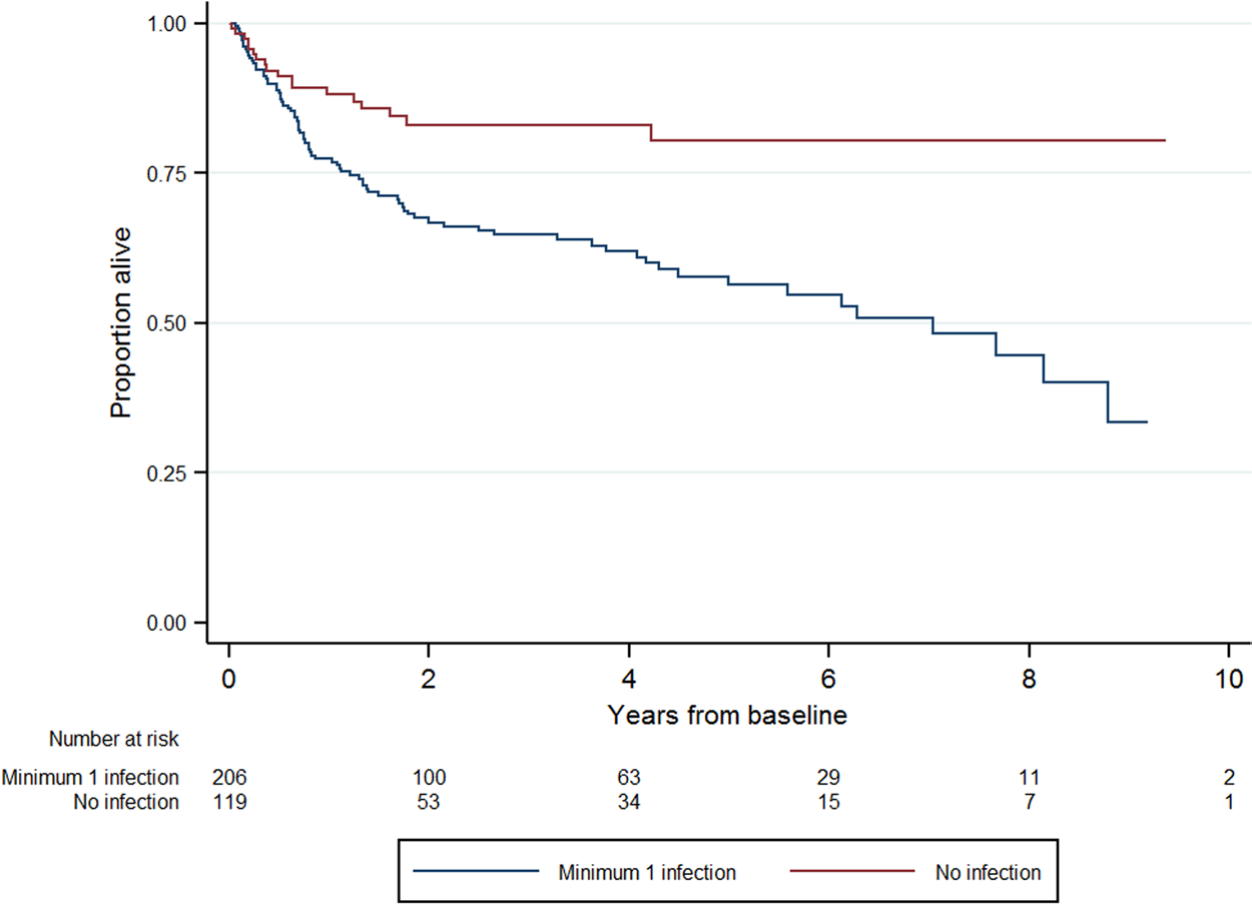
Survival analysis of DLBCL patients who had at least one infectious episode compared with those who did not have an infectious episode.

After adjustment for all other model covariates, factors which remained significant predictors of overall survival in the multivariable analysis were age, Charlson comorbidity score of three or greater (reference category 2 or less), ECOG status of one, three or four (with zero reference category), and an infectious episode (Fig. 1). Chemotherapy cycle number greater than or equal to three was associated with a reduced risk of death compared with cycle number one and two. The presence of neutropenia was associated with reduced survival (adjusted HR 1.95; 95% CI, 1.01–3.78; p = 0.047) compared with no neutropenia.

The regression analysis for predictors of survival was also performed including only patients who received R-CHOP on a 21 day cycle and excluding patients who received R-CHOP like therapy or R-CHOP on a 14 day cycle. See Table 7.

**Table 7:** Regression analysis of the factors associated with survival in patients who received R-CHOP 21 (n = 286).

		Univariate analysis		Multivariable analysis	
		Hazard Ratio	95% CI p value	Hazard Ratio	95% CI p value
Age	<65 years	1.00		1.00	
	>65 years	2.21	1.42 to 3.42 p = <0.001	1.32	0.75 to 2.30 p = 0.328
Sex	Female	1.00		1.00	
	Male	1.19	0.77 to 1.82 p = 0.421	1.49	0.91 to 2.44 p = 0.112
Charlson Comorbidity Score	1–2	1.00		1.00	
	3–5	5.14	3.03 to 8.74 p = <0.001	4.12	2.32 to 7.33 p = <0.0001
	6+	13.88	7.03 to 27.41 p = <0.001	11.02	4.99 to 24.34 p = <0.0001
ECOG	0	1.00		1.00	
	1	4.07	1.69 to 9.78 p = <0.002	2.69	1.00 to 7.26 p = 0.050
	2	8.30	3.46 to 19.89 p = <0.001	2.97	1.27 to 12.41 p = 0.018
	3 and 4	19.83	7.49 to 52.64 p = <0.001	7.16	2.04 to 25.06 p = 0.002
Stage	1	1.00		1.00	
	2	1.29	0.51 to 3.24 p = 0.583	1.25	0.44 to 3.54 p = 0.665
	3	1.56	0.58 to 4.16 p = 0.37	1.17	0.365 to 3.80 p = 0.78
	4	2.43	1.04 to 5.66 p = 0.039	1.22	0.399 to 3.73 p = 0.72
NCCN IPI	Low risk			1.00	
	Low intermediate	1.00		1.00	
	High intermediate	1.35	0.80 to 2.27 p = 0.256	0.88	0.39 to 1.99 p = 0.76
	High	2.61	1.54 to 4.40 p = <0.001	1.82	0.63 to 5.22 p = 0.26
Number of chemotherapy cycles	1–2	1.00			
	3–4	0.13	0.062 to 0.27 p = <0.0001	0.27	0.11 to 0.68 p = 0.005
	5–6	0.08	0.04 to 0.15 p = <0.0001	0.09	0.041 to 0.19 p = <0.0001
	>6	0.10	0.03 to 0.34 p = <0.0001	0.11	0.03 to 0.40 p = <0.0001
Creatinine	Normal	1.00		1.00	
	Raised	1.27	0.82 to 1.96 p = 0.270	0.79	0.44 to 1.43 p = 0.45
Neutropenia within 48 hours of admission with infection	No	1.00		1.00	
	Yes	4.92	2.82 to 8.58 p = <0.0001	3.15	1.66 to 5.96 p = <0.0001
Pegfilgrastim w/I 21 days of admission with infection	No	1.00		1.00	
	Yes	0.82	0.50 to 1.10 p = 0.449	1.26	0.72 to 2.21 p = 0.40
Admission with infection	No	1.00		No	1.00
	Yes	1.63	1.94 to 6.65 p = <0.0001	3.27	1.00 to 2.63 p = <0.046

ECOG = Eastern Cooperative Oncology Group performance status point scale.

NCCN-IPI = International Prognostic Index.

## Discussion

The most notable findings of this study are that infections are common among DLBCL patients receiving R-CHOP and R-CHOP-like chemotherapy and that an infection was associated with reduced overall survival.

The rate of infection in admitted episodes in our population was 63% and of the patients that experienced an infectious episode, 60% experienced multiple episodes. This rate is higher than in other reports, and may be explained by the study design, which included all DLBCL patients undergoing therapy, compared with carefully selected patient populations that are included in clinical trials. Data from observational cohorts have demonstrated higher rates of infection compared with randomised controlled trials with reported rates ranging from 10 to 42%^6–13^. Our higher rates may also be due to differing definitions of infection and/or data collection methods. For example, the risk of an episode of neutropenic fever during R-CHOP chemotherapy has been reported as 19%^4, 5^ however non-neutropenic infective episodes were not documented. In this study the definition of neutropenia was 1.0 × 10^9^/L, while another common definition is 0.5 × 10^9^/L. This may be another explanation for why the neutropenia infection rate on our study was higher than reported elsewhere. Despite differences in definitions, our study suggests rates of infection for DLBCL may be higher in a real world setting and that infection prevention is a key strategy in the supportive management of DLBCL. The results from this study may inform the use of infection prevention strategies, including which patients are most likely to benefit. This study identified patients at high-risk of infection, highlighted the highest risk period during R-CHOP therapy, and provided data on the most common types of infections. Patients with newly diagnosed DLBCL who are at highest risk of infection are those who, have multiple comorbidities, poor performance status and an advanced risk NCCN-IPI. The presence of multiple comorbidities and poor performance status were also predictors of earlier death. Findings from previous studies that describe predictors of infection are inconsistent and use heterogeneous definitions, making it difficult for clinicians to accurately predict the risk of infection in their patients. In the pre-rituximab era, Lyman *et al*. constructed a predictive model that demonstrated, age, LDH, albumin, neutropenia and bone marrow involvement predicted hospitalisation for life threatening neutropenia fever^13^. Pettengell *et al*. found that older age, low albumin, previous chemotherapy and recent infection were predictive of neutropenia fever in cycle one^14^.

In our study, patients were more likely to die from all causes during their first two cycles of chemotherapy compared with subsequent cycles, which is consistent with other studies in lymphoma patients^14, 15^. This suggests that preventative measures could be maximised early in the R-CHOP treatment course, rather than instituted after infection has occurred.

Current strategies to prevent infection include patient education, vaccination, and antimicrobial prophylaxis. In this study, the leading site of infection was the lower respiratory tract. *Streptococcus pneumo*niae is known to cause the majority of these infections^16^ however studies regarding the efficacy of vaccination before the commencement of R-CHOP are lacking. Further research is required to examine the optimal timing, efficacy and clinical outcomes are of pneumococcal vaccination specifically in patients receiving R-CHOP.

In terms of the use of antimicrobial prophylaxis, it is difficult to draw conclusions or make firm recommendations based on the microbiological data acquired through clinical coding data, as non-clinically relevant isolates may have been included. Fungal infections accounted for 19% of infections, which is substantially higher than in other literature^17^. In this study, 2.5% had *Pneumocystis jirovecci*, which is below the 3.5% rate for which prophylaxis is recommended according to Australian national consensus guidelines^18^.

The use of growth factors, such as pegfilgrastim, to reduce the impact of neutropenia is also used to prevent infections. This study confirmed that neutropenia was a strong a predictor of an infectious episode and was associated with reduced survival. The use of pegfilgrastim was also independently associated with a reduction in the risk of an infectious episode. Interestingly, pegfilgrastim use had no significant effect on survival. This is consistent with other studies that have demonstrated reduced risk of severe neutropenia and neutropenia fever with colony stimulating factors but no effect on mortality^15, 19^. Importantly, as our study was a retrospective cohort study, the use of pegfilgrastim was not random and may be a surrogate measure of other factors. International guidelines^4, 20, 21^ recommend primary prophylaxis with colony stimulating factors when the incidence of neutropenia fever is greater than 20% for the chemotherapy regimen. In lymphoma specifically, it is suggested to administer primary prophylaxis in patients older than 65 with comorbidities^4^. Our study would support this recommendation.

The main limitations of this study were that it was performed at a single centre, was retrospective, and relied upon administrative datasets. Use of an administrative dataset may result in missing data or misclassification, which could inaccurately represent the number and type of infections. In addition, patients with infections not requiring admission to hospital are not included. This may underestimate the rate of infections as well as influence whether infection is a predictor of survival when these infections are included. Relying on ICD-10 diagnostic codes to classify the types of organisms may be misleading. Nonetheless, this study represents one of the largest cohorts of DLBCL patients and one of the few studies in real world setting.

Our study has identified a subset of patients at high risk of infection and death and some possible strategies to mitigate this risk. Further research could be directed towards prospectively studying preventative strategies in high-risk patients as identified in this study, with a view to developing preventative strategies that are personalised, targeted and effective.

## Methods

### Study design and setting

A retrospective cohort study was performed at a Monash Health, a 2000 bed academic health service in Melbourne, Australia. All patients with a new diagnosis of DLBCL who received R-CHOP or R-CHOP-like chemotherapy over a 10-year period between 2004 and 2014 were identified using hospital admission data and medical record review.

### Data sources

Demographic data collected from medical records included age, sex, lymphoma diagnosis details (including date, stage and type), Eastern Cooperative Oncology Group (ECOG) performance status classified on a five-point scale^22^, and International Prognostic Index (NCCN-IPI)^3, 23^. Details on chemotherapy regimen, number of cycles, date of death or last follow up was obtained from the medical record.

Data on all hospital admissions for each patient was obtained from the clinical information services, and included admission and discharge dates, diagnostic codes (classified according to the Australian modification of the International Statistical Classification of Diseases and Related Health Problems, Tenth Revision (ICD-10-AM) and procedure codes (classified according to the Australian Classification of Health Interventions)^24^.

The use of colony stimulating factors was obtained from the pharmacy information system. Pathology results were obtained from the pathology laboratory information system.

### Definitions

Co-morbidities were identified using the ICD-10-AM diagnostic codes in admission data and classified according to the Charlson comorbidity index^25^.

The absolute neutrophil count, 48 hours before or after the first day of each admission episode, was identified where available. If the neutrophil count within 48 hours of the admission and including the day of admission was less than 1.0 × 10^9^/L and the admission contained an infectious code, then this was defined as infection with neutropenia. If the neutrophil count within 48 hours of the admission was greater than 1.0 × 10^9^/L and the admission contained an infectious code, then this was defined as infection without neutropenia. If the neutrophil count within 48 hours of the admission was less than 1.0 × 10^9^/L and the admission did not contain an infectious code, then this was defined as neutropenia without infection.

For each admission, the use of pegfilgrastim as primary or secondary prophylaxis within 21 days of the first day of the admission was recorded. At our institution pegfligrastim is used for primary prophylaxis in DLBCL patients aged 65 years or older. Filgrastim use was not considered in the analysis of factors associated with infection, as in our institution it is more frequently administered to patients with established infection rather than as prophylaxis.

#### Infectious outcomes

Infectious episodes were defined as any hospitalization after the date of DLBCL diagnosis with an infection code recorded in the hospital admission data.

Each infection was classified according to body site; blood stream infection (BSI), upper respiratory tract, lower respiratory tract, cardiovascular, gastrointestinal, urogenital, neurological, skin and soft tissue, bone and joint, other, device or line related and source unknown.

For each infection, intensive care unit (ICU) admission, ICU length of stay and the timing of infection in relation to first diagnosis of DLCBL were identified using the admission data.

#### Statistical analysis

Descriptive statistics were used for incidence of infection, types of infection and changes over time. Categorical variables were summarized using frequency and percentage. Continuous variables were summarized using mean and standard deviation (SD) or median and inter-quartile range (IQR) as appropriate.

Conditional risk set time-to-event modeling for multiple failure time data was used to determine possible clinical predictors of infection using episodes of infection as the evaluable outcome. In this model, subjects were permitted to contribute multiple events (infection episodes) to the analysis. Due to the multiplicity of events, the model considers the entire time period at risk of infection (period of patient follow-up) for the specified outcome of interest, rather than censoring a patient at the first observed infection event. In the survival analysis, the proportion of patients with neutropenia and infection was compared to the proportion of patients with neutropenia without infection and the proportion of patients with pegfilgrastim use and infection was compared to the proportion of patients with pegfilgrastim use without infection.

A Cox proportional hazards regression was used to investigate predictors of mortality. For both models, hazard proportionality was analyzed using analysis of scaled Schoenfeld residuals. For all analyses p < 0.05 was considered significant. All analyses were performed using Stata version 14, (StataCorp Inc., College Station, TX, USA).

The project was approved by the Monash Health Human Research Ethics Committee. All methods were carried out in accordance with relevant guidelines and regulations.

### Data Availability

The datasets generated during and analysed during the current study are not publicly available due to patient confidentiality but are available from the corresponding author on reasonable request.
